# Consider cerebral tuberculosis as differential of SARS-CoV-2-associated acute, haemorrhagic, necrotising encephalitis

**DOI:** 10.1186/s41983-022-00506-5

**Published:** 2022-06-03

**Authors:** Josef Finsterer, Daniel Matovu, Ana C. Fiorini, Fulvio A. Scorza

**Affiliations:** 1 Neurology & Neurophysiology Center, Postfach 20, 1180 Vienna, Austria; 2 Disciplina de Neurociência, Universidade Federal de São Paulo/Escola Paulista de Medicina (UNIFESP/EPM), Rua Pedro de Toledo, 697 - Vila Clementino, São Paulo, SP 04039-00 Brazil; 3 Programa de Estudos Pós-Graduado Em Fonoaudiologia, Pontifícia Universidade Católica de São Paulo (PUC-SP), São Paulo, Brazil; 4 Departamento de Fonoaudiologia, Escola Paulista de Medicina/Universidade Federal de São Paulo (EPM/UNIFESP), R. Botucatu, 740 - Vila Clementino, São Paulo, SP 04023-062 Brazil

## Letter to the Editor

With interest we read the article by Ermilov et al. about a young male in his twenties, born to a consanguineous marriage, who developed a spontaneous pneumothorax after having been hospitalised in a primary infectious hospital for moderate COVID-19 [1]. Work-up for the cause of pneumothorax revealed “infiltrative pulmonary tuberculosis in the phase of disintegration and seeding” [1]. Consecutively the patient developed impaired consciousness and epilepsy which was attributed to fatal acute, haemorrhagic, necrotising encephalitis (AHNE) [1]. It was concluded that “the death of the patient, as well as the severity of the disease, was largely due to COVID-19”. The study is appealing, but raises concerns which require comments.

We do not agree with the notion that SARS-CoV-2 was definitively responsible for AHNE. As long as cerebral tuberculosis had not been appropriately excluded, as long as SARS-CoV-2 was not documented in the CSF or the cerebrum, and as long as upregulation of cytokines, chemokines, and glial markers had not been documented in the CSF, a causal relation between AHNE and SARS-CoV-2 remains speculative. Arguments against SARS-CoV-2 as the cause of AHNE are that in a recent review about the neurological and neuroimaging findings of 584 patients with COVID-19, AHNE has not been mentioned [2] and that AHNE had only been rarely reported in association with SARS-CoV-2 [3].

Regarding cerebral tuberculosis, neither the cerebro-spinal fluid (CSF) intra vitam nor the brain at autopsy had been investigated for *Mycobacterium tuberculosis* by culture, histology, immune histology, or PCR [1]. Excluding cerebral tuberculosis is crucial given the findings of generalised cerebral vasculitis, which occurs in up to one-quarter of the patients with intra-cranial tuberculosis [4].

Missing is the visualisation of the cerebral vessels by conventional angiography, computed tomography angiography (CTA), or by magnetic resonance angiography (MRA) [1]. Since the patient presented with generalised cerebral vasculitis on autopsy [1], it would have been useful to document cerebral vasculitis on imaging of the cerebral vasculature. It would have been also necessary to exclude aneurysm formation, which can be a complication of tuberculous cerebral vasculitis [5].

A further limitation is that no explanation of the cause of tremor three years prior to admission had been provided. Since tuberculosis is a chronic disease, it is conceivable that tremor was already a clinical manifestation of cerebral tuberculosis. It is also conceivable that tremor was in fact a focal seizure given the occurrence of focal seizures during hospitalisation.

Though the figure caption of figure-1 promises to show a diffusion-weighted imaging (DWI), only T2/TIRM images are presented. Missing is also a susceptibility-weighted imaging (SWI) to document the bleeding. Furthermore, an explanation for the narrowing of the subarachnoid space on MRI should be provided. We should be told if this was an artefact, due to meningitis, or due to cerebral oedema. Missing is the treatment the patient received for tuberculosis.

Overall, the interesting study has several limitations which challenge the results and their interpretation. Attributing AHNE to the SARS-CoV-2 infection is speculative as long as cerebral tuberculosis has not been convincingly excluded.

## Data Availability

All data reported are available from the corresponding author.

## Abbreviations

AHNE: Acute, haemorrhagic, necrotising encephalitis
CSF: Cerebro-spinal fluid
CTA: Computed tomography angiography
DWI: Diffusion-weighted imaging
MRA: Magnetic resonance angiography
SWI: Susceptibility-weighted imaging
